# Prognostic Risk Factors of Oral Squamous Cell Carcinoma: A Retrospective Case-Control Study at a Tertiary Hospital in Riyadh, Saudi Arabia

**DOI:** 10.7759/cureus.84213

**Published:** 2025-05-16

**Authors:** Nasser M AlMadan, Abdulrahman AlHamidi, Bashayer AlMalki, Shahad Aladhyani, Maha Ali Al-Mohaya, Mai Almohaya, Assem S Alrumeh

**Affiliations:** 1 Department of Dentistry, Ministry of Health, Hafar Al-Batin, SAU; 2 Department of Dentistry, Prince Sultan Military Medical City, Riyadh, SAU; 3 Department of Oral Medicine and Special Care Dentistry, Prince Sultan Military Medical City, Riyadh, SAU; 4 Department of Dentistry, Iman General Hospital, Riyadh, SAU; 5 Department of Pathology and Laboratory Medicine, Prince Sultan Military Medical City, Riyadh, SAU

**Keywords:** oral cancer, oral squamous cell carcinoma, prognostic factors, squamous cell carcinoma, survival analysis

## Abstract

Background

Oral squamous cell carcinoma (OSCC) is the most prevalent malignant tumor in the oral cavity, often associated with poor prognosis, particularly in advanced stages. While smoking, alcohol consumption, and areca nut use are recognized risk factors globally, shamma (Arabian snuff) stands out as a significant risk factor specific to Saudi Arabia.

This study aims to evaluate the clinical and demographic factors that influence outcomes in patients diagnosed with OSCC.

Methodology

This retrospective case-control study was performed at Prince Sultan Military Medical City in Riyadh, Saudi Arabia, from January 2010 to December 2022. It included all cases diagnosed with OSCC that received treatment and follow-up at our institution. Clinical and demographic data were collected from electronic medical records and pathology requests. Statistical analysis, including descriptive statistics, frequency distribution, survival analysis with the Kaplan-Meier method, and regression analysis with the Cox proportional-hazards regression method, was performed using SPSS version 24 (IBM Corp., Armonk, NY).

Results

Eighty-eight cases were included in the study, with 52 (59.1%) occurring in males. The most common tumor site was the lateral tongue, accounting for 55 cases (62.5%), followed by the buccal and alveolar mucosa, each with 8 cases (9.1%). Shamma was the most frequently reported risk factor, present in 21 cases (23.9%), followed by tobacco smoking in 14 cases (15.9%). Death was reported in 33 patients at the end of the study period (37.5%), with a five-year overall survival rate was 59.9%, and the total overall survival rate was 38.7%, with a mean survival time of 95 months (95% confidence interval (CI) 79.5-110). Older age (more than 40 years), high stage, and presence of lymph node metastasis were significantly associated with the worst overall survival, while negative margin was significantly related to an improved overall survival.

Conclusions

OSCC is a highly aggressive malignancy with poor outcomes. Factors such as older age, advanced stage, and lymph node metastasis are linked to worse overall survival.

## Introduction

Oral squamous cell carcinoma (OSCC) is a prevalent malignant neoplasm with poor outcomes, particularly in advanced stages. OSCC ranks as the sixth most common cancer in men and the fifteenth in women, accounting for approximately 94% of all oral malignancies. A major contributor to the high mortality rate associated with oral cancer is that 60% of patients are diagnosed at an advanced stage. The global distribution of OSCC varies owing to different etiological factors. OSCC presents in various forms, with some cases identified late, resulting in a poor prognosis [1]. OSCC is the most common head and neck cancer worldwide, with an incidence of 377,713 cases in 2020, the majority of which were in Asia (248,360), followed by Europe (65,279) and North America (27,469). The five-year prevalence of OSCC nearly reached 1 million (959,248 cases), with most cases in Asia, followed by Europe and North America [2]. Prevalence varies by country, with South Asian nations-particularly India, Pakistan, Bangladesh, and China-showing a high incidence of OSCC [3]. In Saudi Arabia, the distribution varied across regions with the highest rate in Jazan (3.5%), followed by Riyadh (2.1%), and Eastern Province (1.8%), and this could be owned to the increasing use of shamma (Arabian snuff) in Jazan region [4-6]. Quadri et al. found that shamma use increased the likelihood of developing OSCC by 37.2-fold (odds ratio (OR) = 37.2; confidence interval (CI): 12.3-113.2) while tobacco smoking increased the likelihood by 10.5-fold (OR = 10.5; CI: 2.88-13.11) [7]. Moreover, the use of shamma leads to changes in anatomical distribution, with the buccal mucosa becoming the second most commonly affected site after the tongue. It also skews the gender distribution, with a higher incidence observed in females [5]. Cases with a history of shamma usage exhibited less deep invasion, no lymph node metastasis, and a better overall prognosis [8].

Prognosis is influenced by various clinical features, including age, tumor location, size, presence of lymph node metastasis, and overall tumor stage. Advanced age and a higher TNM stage are associated with poorer treatment outcomes. Tumor location also affects survival rates, with lip tumors having better outcomes owing to earlier detection and lower clinical stages, whereas tumors in the floor of the mouth, tongue, and palate have the worst prognosis [9]. In Saudi Arabia, a study performed at Prince Sultan Military Medical City found that tumor stage is a strong predictor of tumor behavior, with advanced stages closely related to tumor progression and cancer-related mortality [10]. This study aims to evaluate the clinical and demographic factors of an OSCC cohort in a tertiary hospital in Riyadh and determine the factors that influence outcomes.

## Materials and methods

This study was conducted per the Declaration of Helsinki, and the protocol of the study was approved by the Institutional Review Board (IRB) of Prince Sultan Military Medical City, Saudi Arabia (IRB: E-2154).

This retrospective, single-center case-control study was conducted on patients who presented to the Head and Neck Oncology Service at Prince Sultan Military Medical City (PSMMC) in Riyadh, Saudi Arabia. The study was performed from January 2010 to December 2022 and included patients diagnosed with OSCC. Cases treated outside our institution, owing to incomplete documentation, and those who did not undergo surgical excision, or cases with missing data were excluded from the study, Clinical records were reviewed, and data were retrieved from medical reports and pathology requests, covering patients' demographics including age and gender with clinical presentation including initial presentation, anatomical location, and tumor staging according to TNM staging protocol [11]. Additionally, we collected comorbidities, habits, treatment methods, and outcomes from the medical electronic files with death as the outcome. Data collection was done by (N.M.), and data validation was done by AH and SA for completeness and accuracy.

Descriptive analyses were used to measure the frequency of key variables, while associations between variables were assessed using the log-rank test; a *P*-value of less than 0.05 was considered statistically significant. Overall survival was calculated from the date of surgery to death (event) or last follow-up (censored) with Kaplan-Meier method was used to estimate the survival curve. The Cox proportional hazards model has been used to analyze the prognostic factors and calculate the hazard ratios (HR).

Results were presented as HRs with 95% CIs with adjustment to confounders, including age, gender, location, cancer history, and tobacco smoking. For the margin, the provision of adjuvant therapy was added. The statistical analysis was performed using SPSS 24 (Released 2016. IBM SPSS Statistics for Macintosh, Version 24.0; IBM Corp., Armonk, NY).

## Results

Eighty-eight patients met our inclusion criteria, with a mean follow-up period of 50.5 months (standard deviation (SD): 37.7). Patient demographic data are detailed in Table 1, showing a male predominance with 52 cases (59.1%) and a mean age of 61.8 years (SD: 15.6; range: 30-91). Males had a slightly higher mean age compared to females, at 62.2 years (SD: 15.7, range: 30-91) versus 61.3 years (SD: 15.7), respectively. The male predilection could be attributed to increased shamma use among males (male: 13, female: 8) and tobacco smoking (male: 13, female: 1).

**Table 1:** Patients’ clinical and demographic data.

Characteristics	*n* (%)
Total cases	88 (100)
Gender	
Male	52 (59.1)
Female	36 (40.9)
Age group	
Less than 40	9 (10.2)
40-65 years	42 (47.8)
More than 65 years	37 (42)
Medical comorbidity	
Hypertension	36 (40.9)
Diabetes mellitus	35 (39.8)
Hypothyroidism	13 (14.8)
Dyslipidemia	11 (12.5)
Asthma	7 (8)
Ischemic heart disease	4 (4.5)
History of stroke	3 (3.4)
Kidney disease	2 (2.3)
Depression	3 (3.4)
Liver disease	1 (1.1)
Anemia	1 (1.1)
Parkinson disease	1 (1.1)
Ataxia	1 (1.1)
Rheumatoid arthritis	1 (1.1)
Interstitial lung disease	1 (1.1)
Cancer history	9 (10.2)
No comorbidity	29 (33)
Habits	
Tobacco smoking	14 (15.9)
Shamma (Arabian snuff) use	21 (23.9)
Alcohol	1 (1.1)
Khat (Bushmen's tea)	1 (1.1)
Region	
South	34
North	4
East	1
West	4
Riyadh	30
Anatomical location	
Lateral tongue	55 (62.5)
Floor of the mouth	5 (5.7)
Buccal mucosa	8 (9.1)
Gingiva	6 (6.8)
Alveolar mucosa	8 (9.1)
Lip	2 (2.3)
Palate	4 (4.5)

Our cohort exhibited a high prevalence of medical comorbidities, with only 29 cases (32.9%) being free of such conditions. Hypertension and diabetes mellitus were present in around 40% of patients, and this might be related to the advanced age of our cohort. Shamma use was the most frequently reported habit, found in 21 cases (23.9%), while smoking was reported in 14 cases (15.9%). A combination of both habits was observed in 6 cases (6.8%).

Pain was the most frequently reported initial symptom, occurring in 40 cases (45.4%), while swelling was reported in 40 cases (45.4%). Additionally, dental pain was the initial symptom in 3 cases (3.4%), and numbness and ear pain were each reported in 1 case (1.1%). Half of the cases presented clinically as ulcerated lesions (44 cases), whereas 10 cases (11.4%) presented as white patches, verrucous lesions were reported in 4 patients (4.5%), and induration was reported 13 times (14.8%).

Table 2 outlines the tumor characteristics and treatment modalities. Most cases were classified as stage 4, with 29 cases (33%), followed by stage 1, which was observed in 27 cases (30.7%). This distribution is related to the delayed diagnosis of our cohort, with a mean time of 8 months (SD: 7.9, range 1-36 months). Surgical resection was performed in all cases, with 81 of these cases also undergoing neck dissection (92%). Neck dissection followed by radiotherapy was reported in 27 cases (30.7%), while neck dissection followed by chemoradiation was used in 39 cases (44.3%).

**Table 2:** Clinical presentations and treatment modalities used in patient management.

Characteristics	*n* (%)
T stage	
T1	30 (34.1)
T2	23 (26.1)
T3	14 (15.9)
T4	21 (23.9)
Node metastasis	
N0	61 (59.3)
N+	27 (30.7)
TNM stage	
Early stage (1+2)	44 (50)
Late stage (3+4)	44 (50)
Surgery	
Yes	88 (100%)
Neck dissection	
Yes	81 (92)
No	7 (8)
Adjuvant radiotherapy	27 (30.7)
Adjuvant chemoradiation	39 (44.3)

Table 3 shows the demographic and clinical characteristics of OSCC secondary to shamma use. Most of the shamma users were from the southern region (18 patients, 85.7%), and the lateral tongue was the most commonly involved site (12 patients, 57.1%). Around three-quarters of the shamma users were diagnosed in the advanced stage (stages 3 and 4), with 38% having lymph node metastasis. Additionally, death was reported in eight patients (38.1%).

**Table 3:** Demographic and clinical characteristics of shamma-related OSCC. OSCC, oral squamous cell carcinoma

Variable	*n* (%)
Total number	21 (100)
Age group	
<40 years	0 (0)
40-65 years	12 (57.1)
>65	9 (42.9)
Gender	
Male	13 (61.9)
Female	8 (38.1)
Tobacco smoking	6 (28.6)
Location	
Lateral tongue	12 (57)
Buccal mucosa	4 (19)
Floor of the mouth	2 (9.5)
Gingiva	1 (4.5)
Lip	1 (4.5)
Alveolar mucosa	1 (4.5)
Region	
South	18 (85.7)
Riyadh	2 (9.5)
West	1 (4.5)
T stage	
T1	4 (19)
T2	5 (23.8)
T3	3 (14.3)
T4	9 (42.9)
Lymph node	
LN0	13 (61.9)
LN+	8 (38.1)
Stage	
Stage 1	3 (14.3)
Stage 2	2 (9.5)
Stage 3	6 (28.6)
Stage 4	10 (47.6)
Margin	
Positive	7 (33.3)
Close	4 (19)
Negative	10 (47.6)
Death	8 (38.1)

Table 4 presents the mean overall survival for patients with OSCC. Out of the total cases, 33 patients (37.5%) died owing to OSCC. The five-year overall survival rate was 59.9%, and the total overall survival rate was 38.7%, with a mean survival time of 95 months (95% CI: 79.5-110).

**Table 4:** Survival analysis for key clinical variables. NR, not reached

Variables			Log-rank test *P*-value	Three-year OS	Five-year OS	OS	Mean OS by months (95% CI)
Age		<40 years	0.022	100%	85.7%	85.7%	121.1 (99.6-142.7)
		40-65 years		79.3%	69.1%	23%	99.1 (77.8-120.3)
		>65 years		67.5%	42.3%	31.7%	72 (50.1-94)
Gender		Male	0.379	80.9%	65.2%	39.9%	99.7 (79.8-119.7)
		Female		73.8%	54.3%	36.2%	85.7 (64.6-106.8)
Tobacco smoking		Yes	0.080	88.9%	88.9%	44.4%	128.4 (99.4-157.5)
		No		75.7%	55.7%	38.3%	86.5 (71.3-101.8)
Shamma		Yes	0.407	74%	59.2%	44.4%	74.6 (51.8-97.4)
		No		79.1%	60.9%	40.8%	98.7 (81.7-115.6)
Anatomical site		Lateral tongue	0.011	78.4%	56.6%	33%	NR
		Floor of the mouth		100%	100%	100%	NR
		Buccal mucosa		85.7%	85.7%	85.7%	NR
		Gingiva		NR	NR	NR	NR
		Alveolar mucosa		87.5%	87.5%	87.5%	NR
		Palate		100%	66.7%	66.7%	NR
		Lip		NR	NR	NR	NR
Treatment modality		No adjuvant therapy	0.029	90.9%	66.3%	66.3%	107.4 (79.9-135)
		Adjuvant radiation		83.2%	73%	62.6%	115.1 (90-140.2)
		Adjuvant chemoradiation		67.1%	47.7%	13.2%	68.7 (51.3-86.1)
Metformin		Yes	0.281	78.8%	71.6%	53.7%	113.3 (85.7-140.8)
		No		76.4%	56.2%	31.4%	86.1 (70.3-102)
Aspirin		Yes	0.132	87.2%	77.5%	51.7%	119.2 (90.1-148.3)
		No		75.4%	66.3%	35.9%	85.9 (70.5-101.3)
Margin		Negative	0.032	85.7%	78.4%	52.6%	109.9 (91.7-128.1)
		Close		82.5%	47.6%	23.8%	83.6 (52.5-114.7)
		Positive		62.2%	42.3%	42.3%	65 (46.2-83.7)
Lymph node		N0	0.04	83.9%	67.1%	50%	105.5 (87.7-123.2)
		N+		63.7%	44.6%	NR	70 (47.6-92.4)
Stage		Low stages (1-2)	0.029	89.7%	70.6%	49.9%	109.6 (90.1-129.1)
		High stages (3-4)		65.9%	50%	NR	74.2 (56.7-91.8)
Overall survival				76.7%	59.9%	38.7%	95 (79.5-110)

**Figure 1:**
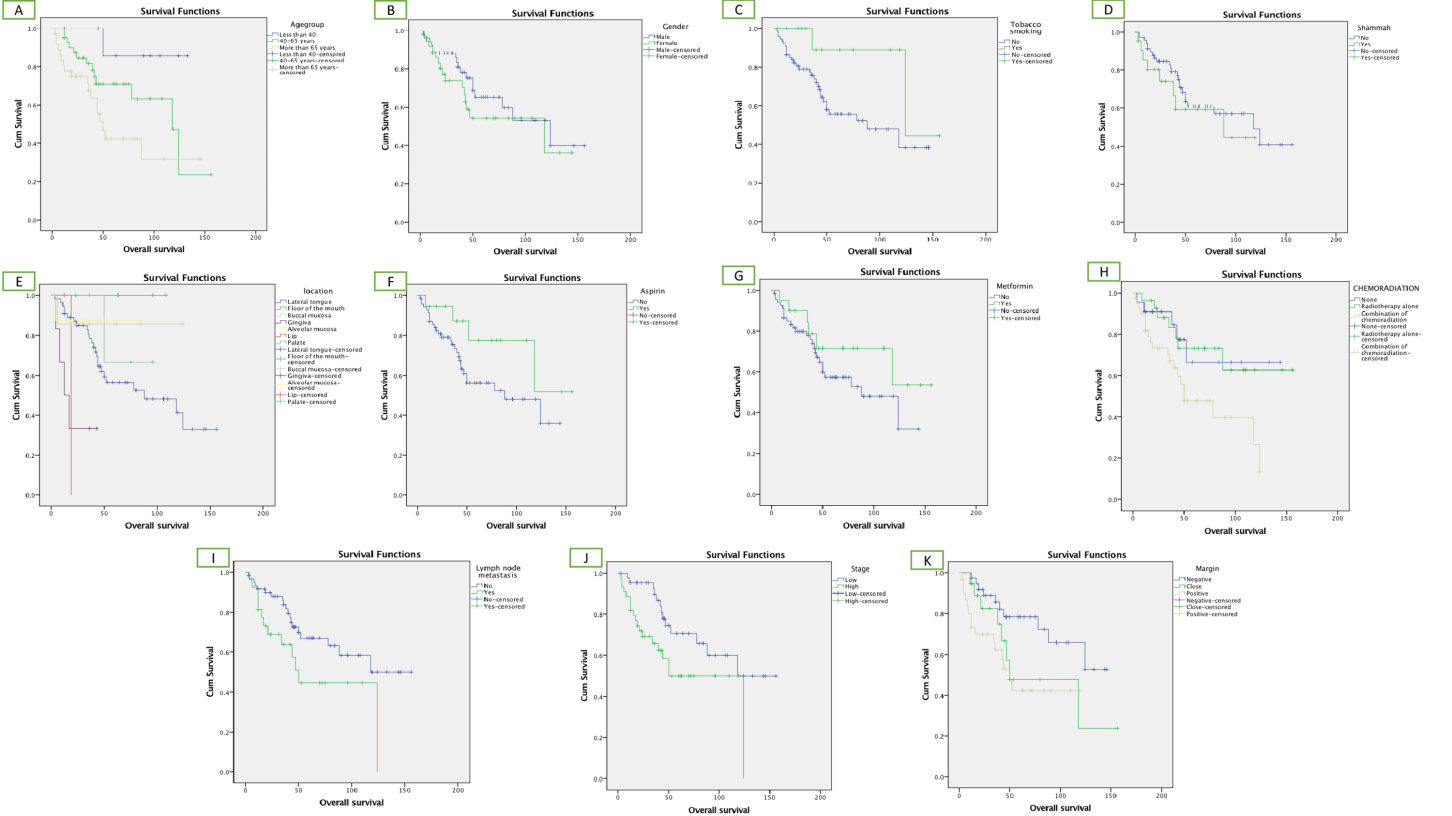
Survival analysis of key variables. Kaplan-Meier curves of multiple demographic and clinical factors including age group (A), gender (B), tobacco smoking (C), shamma use (D), anatomical location (E), aspirin use (F), metformin use (G), use of adjuvant therapy (H), presence of lymph node metastasis (I), clinical stage (J), and margin status (K).

Older age (over 65) was statistically associated with poorer survival outcomes, with a *P*-value 0.022 (72 months; 95% CI: 50.1-94), while lower stage (stages 1-2) was statistically associated with better overall stage with *P*-value 0.029 (100.6 months; 95% CI: 90.1-129.1). Additionally, the absence of lymph node metastasis was correlated to better overall survival with a *P*-value of 0.04 (105.5 months; 95% CI: 87.7-123.2). Similarly, negative margin was related to better overall survival with a *P*-value of 0.032 (109.9 months; 95% CI: 91.7-128.1). Notably, patients using metformin and aspirin showed improved overall survival; however, it was not statistically significant (Figure 1). 

Table 5 shows the hazard ratios (HRs) calculated using the Cox proportional hazards model. Older age was associated with an increased risk of death (HR: 2.108, 95% CI: 1.197-3.713), which was statistically significant (*P* = 0.01). Presence of lymph node metastasis and higher stage were independent risk of worse outcomes (HR: 2.711, 95% CI: 1.199-6.130; HR: 4.589, 95% CI: 1.783-11.807), while negative margin was an independent risk of better outcomes (HR: 0.246, 95% CI: 0.101-0.601).

**Table 5:** Hazard ratio for the key variables. HR, hazard ratio; CI, confidence interval

Factor			Crude HR (95% CI)	*P*-value	Adjusted HR (95% CI)	*P*-value
Gender	Female		1	0.382	Not done	
	Male		0.733 (0.366-1.470)			
Age group	<40 years		1	0.010	Not done	
	40-65 and >65		2.108 (1.197-3.713)			
Shamma use	No		1	0.411	Not done	
	Yes		1.402 (0.626-3.141)			
Tobacco smoking	No		1	0.1	Not done	
	Yes		0.299 (0.071-1.259)			
Metformin	No		1	0.287	Not done	
	Yes		0.615 (0.251-1.506)			
Aspirin	No		1	0.143	Not done	
	Yes		0.456 (0.159-1.303)			
Margin	No		0.351 (0.155-0.795)	0.012	0.246 (0.101-0.601)	0.002
	Close		0.737 (0.310-1.748)	0.488	0.340 (0.131-0.885)	0.027
	Positive		1		1	
Lymph node metastasis	N0		1	0.045	1	0.017
	N+		2.070 (1.016-4.219)		2.711 (1.199-6.130)	
Stage	Low stages (1-2)		1	0.033	1	0.002
	High stages (3-4)		2.177 (1.063-4.457)		4.589 (1.783-11.807)	

## Discussion

95% CI:OSCC is the most common malignancy in the oral cavity, with a multifactorial etiology. It predominantly affects the elderly population. Tobacco use, whether smoking or chewing, is a significant risk factor for developing oral cancer. Other risk factors include alcohol consumption and human papillomavirus (HPV) infection. OSCC has the potential to invade underlying bone and involve nerves. Metastasis significantly affects the five-year survival rate, with approximately 50% of patients with head and neck squamous cell carcinoma experiencing recurrence and metastasis in the first two years [12].

In Saudi Arabia, the incidence of OSCC is 2.3 per 100,000, with a slightly higher rate in males compared to females. This disparity may be attributed to the higher prevalence of smoking among males, with a ratio of 15.8:1 [13,14]. Our study also shows a male predominance, with 52 cases (59.1%), likely owing to higher rates of shamma and tobacco smoking among males in our cohort (13 cases each of shamma and tobacco smoking) compared to females (8 cases of shamma and 1 case of tobacco smoking). These findings are consistent with reports from the United States [15], the United Kingdom [16], Spain [17], China [18], and Taiwan [19], although higher female prevalence has been noted in Thailand [20]. The mean age of our cohort was 61.8 years (SD: 15.6; range: 30-91). Nine cases (10.2%) were observed in patients younger than 40, including 5 cases in males. A recent multinational study identified 626 cases of OSCC in young patients (under 40 years) out of 10,727 total cases (5.8%), with 358 of these cases in males (57.2%). Our results exceed the cumulative percentage reported by all countries except for the one in India, which reported 13.2% of their cases in patients younger than 40. This higher percentage in India may be related to the high consumption of areca nuts in that population [21]. Interestingly, only one patient younger than 40 years in our cohort had a known risk factor (smoking), suggesting that genetic factors may play a role, particularly considering the prevalence of consanguineous marriages in our population. 

Medical comorbidities were reported in 67.4% of our cohort, with hypertension occurring in 40.4%, considerably higher than the national prevalence of 22.66% [22]. Diabetes mellitus (DM) was present in 39.3% of cases, compared to the national prevalence of 28% [23]. Shamma use was the most commonly reported habit, seen in 21 cases (23.9%), while tobacco smoking was reported in 14 cases (15.9%). Only six patients used both shamma and tobacco (6.8%). Alcohol and khat (Bushmen's tea) use were noted in just one case each (1.1%). This demographic distribution is noteworthy because tobacco smoking was not the predominant risk factor for OSCC as commonly reported worldwide, and alcohol use was much less prevalent compared to other regions [24,25].

Half of the cases were diagnosed at advanced stages (stages 3 and 4), with 27 cases (30.7%) presenting initially with lymph node metastasis. The delay in diagnosis may be attributed to our institute's role as a tertiary hospital serving patients from Riyadh and surrounding areas, which can contribute to longer diagnostic delays. Additionally, delays may be related to the time it takes for patients to recognize their symptoms or a lack of awareness among healthcare providers about this type of lesion [26,27]. The COVID-19 pandemic and the associated quarantine and lockdown measures could also have contributed to the diagnostic delays observed in our cohort [28].

All cases underwent surgical resection followed by cervical neck dissection, except 7 cases (8%). Adjuvant radiotherapy was administered to 27 cases (30.7%), while 39 cases (44.3%) received adjuvant chemoradiotherapy. Of the 88 cases, 33 patients died by the end of the study, representing 37.5% of the cohort. The five-year overall survival rate was 59.9%, while the overall survival rate at the end of the study was 38.7% (95 months, 95% CI: 79.5-110). Al-Jaber et al. found that the five-year overall survival for oral cancer in multiple Arab countries ranged from 20% to 59.4%, which is consistent with our findings [29]. Additionally, a previous study performed at our institute covering patients from 2009 to 2015 reported a five-year overall survival rate of 58.13%, suggesting no improvement in survival rates for OSCC cases [10].

Older age was associated with poorer overall survival, with 5-year overall survival of 42.3% and 31.7% overall survival (72 months; 95% CI: 50.1-94). Overall survival rates were similar between genders.

Interestingly, patients who used shamma had worse outcomes (HR: 1.402, 95% CI: 0.626-3.141); however, it was not statistically significant with a *P*-value: 0.411, which contrasts with the findings of Al-Balawi and Nwoku, who reported better outcomes for shamma users [8]. Conversely, tobacco smoking was associated with a much better prognosis (HR: 0.299, 95% CI: 0.071-1.259), with a five-year overall survival rate of 88.9%. This finding contradicts the majority of studies in the literature, which generally report worse overall survival for smokers [30-32]. Our results can be explained by the low prevalence of alcohol consumption in our population because alcohol has a synergistic effect with tobacco smoking and can increase disease aggressiveness [17].

Advanced stage (stages 3-4) and the presence of lymph node metastasis were independent risk factors for more aggressive outcomes, both reaching statistical significance (HR: 4.589; 95% CI: 1.783-11.807 and HR: 2.711; 95% CI: 1.199-6.130, respectively), with *P*-values 0.002 and 0.017, respectively. In contrast, negative margins were related to worst overcome (HR: 0.246; 95% CI: 0.101-0.601) and were statistically significant with a *P*-value of 0.002.

Use of metformin and aspirin was associated with improved five-year overall survival rates of 71.6% and 77.5%, respectively, which agrees with existing literature that demonstrates survival benefits for patients using these medications [33,34]. This may be attributed to metformin's role in suppressing tumor cell proliferation, inducing apoptosis, and reducing the risk of metastasis [35].

Study limitations

Our study has several limitations, including its retrospective design, the small sample size from a single tertiary center, lack of knowledge of the duration and extent of smoking and shamma habits, and no information regarding socioeconomic status. 

Recommendations and future directions

We recommend performing multicenter prospective studies across different regions of the country. Additionally, the incorporation of the histological features with clinical data to better assess prognostic factors for this patient cohort is undergoing.

## Conclusions

OSCC is a highly aggressive malignancy with poor outcomes. Early diagnosis and management are essential for improved outcomes, as advanced stage and presence of lymph node metastasis are independent risk factors of disease outcome. Additionally, it is important to remove the tumor with a free margin, as an involved margin is associated with the worst survival. 
